# Linking Mitochondrial Dysfunction to the Immune Microenvironment in HFpEF: An Integrated Bioinformatics and Experimental Approach

**DOI:** 10.1002/iid3.70440

**Published:** 2026-04-22

**Authors:** Yingying Xie, Haoming He, Yike Li, Qiang Chen, Yanxiang Gao, Jingang Zheng

**Affiliations:** ^1^ Department of Cardiology, China‐Japan Friendship Hospital (Institute of Clinical Medical Sciences) Chinese Academy of Medical Sciences & Peking Union Medical College Beijing China; ^2^ Department of Cardiology The Second Xiangya Hospital of Central South University Changsha China; ^3^ Department of Cardiology, China‐Japan Friendship Hospital Chinese Academy of Medical Sciences & Peking Union Medical College Beijing China

**Keywords:** bioinformatics analysis, Heart failure with preserved ejection fraction, Hub Mito‐DEGs, Immune infiltration, Mitochondrial dysfunction

## Abstract

**Background:**

The interplay between mitochondrial dysfunction and immune infiltration in heart failure with preserved ejection fraction (HFpEF) remains poorly understood. This study aimed to elucidate this relationship and identify key regulatory genes.

**Methods:**

We integrated bioinformatics analyses of human HFpEF datasets (GSE108904, GSE126062) with experimental validation. Differential expression analysis, weighted gene co‐expression network analysis (WGCNA), and the MitoCarta3.0 database were used to identify hub mitochondrial genes. Immune cell infiltration was assessed, and key findings were validated in peripheral blood mononuclear cells (PBMCs) from HFpEF patients and in mouse/cell models of the disease.

**Results:**

Two hub mitochondrial genes, CHCHD1 and EFHD1, were associated with HFpEF. Immune profiling revealed increased macrophage infiltration in HFpEF, which was negatively correlated with the expression of both hub genes. Experimental validation confirmed a significant decrease in EFHD1 expression in HFpEF patient PBMCs, HFpEF mouse heart tissues, and a cellular inflammation model. Critically, EFHD1 expression showed a significant negative correlation with clinical indicators of heart failure (NT‐proBNP and E/e' ratio).

**Conclusion:**

Our integrated analysis reveals CHCHD1 and EFHD1 as key mitochondrial genes linking mitochondrial dysfunction to immune dysregulation in HFpEF, offering new insights into its molecular mechanisms and potential therapeutic targets.

## Summary

- Identified two key Mito‐DEGs, CHCHD1 and EFHD1, that are associated with HFpEF.
- Correlation between CHCHD1 and EFHD1 and immune infiltration was evaluated using ImmuCellAI.
- CHCHD1 and EFHD1 were negatively associated with macrophages and their subtypes.
- Potential therapeutic agents for CHCHD1 and EFHD1 were predicted using CTD database.
- Targeted EFHD1 immunotherapy may improve the prognosis of patients with HFpEF.

## 1 Introduction

Heart failure (HF) is a major global health challenge affecting approximately 64 million people worldwide, causing high rates of hospitalization and mortality [1]. In China, HF prevalence is about 1.3% among adults aged ≥ 35 years, representing nearly 8.9 million patients [2]. Heart failure with preserved ejection fraction (HFpEF) has emerged as the predominant subtype, accounting for about 50% of all HF cases [3]. According to the Chinese Heart Failure Center Registry, HFpEF patients face poor outcomes, with 1‐year all‐cause readmission and mortality rates of 22.2% and 8.5%, respectively [4]. Given this substantial national and regional disease burden, and the current lack of effective therapies, there is an urgent need to elucidate HFpEF mechanisms and identify novel therapeutic targets relevant to affected populations in China.

Myocardial mitochondria serve as the primary source of energy for cardiac metabolism [5]. In normal physiological conditions, mitochondria play a crucial role in energy generation, maintenance of electrolyte balance, regulation of cell apoptosis, and calcium signaling in myocardial cells [6, 7, 8]. Mitochondrial dysfunction is a well‐recognized pathophysiological mechanism in HF [9]. Patients with HFpEF exhibit structural and energy‐related mitochondrial irregularities in myocardial cells [10]. These alterations may stem from various pathophysiological processes, including heightened mitochondrial free radical damage that results in decreased ATP production [11]. Changes in ATP levels impact the balance between myocardial cell supply and demand, triggering the activation of downstream signaling pathways that often lead to inflammation and diastolic dysfunction [12].

Recently, there has been a growing interest in immune infiltration within the realm of cardiovascular diseases [13]. Prolonged damage to cardiomyocytes has been noted to result in heightened apoptosis and the initiation of chronic inflammation characterized by the infiltration of monocytes and lymphocytes [14]. Nevertheless, there remains a dearth of knowledge regarding the scope of immune infiltration in HFpEF.

Bioinformatics plays a pivotal role in facilitating the shift from conventional medicine towards predictive, preventive, and personalized medicine [15]. The present study utilized bioinformatics to explore the contribution of genes related to mitochondria in the progression of HFpEF and their association with immune infiltration. This investigation aims to enhance comprehension of the fundamental immunometabolism processes and bolster the development of predictive/diagnostic instruments and therapeutic interventions.

## 2 Methods

### 2.1 Ethical Statement

#### 2.1.1 Patients

This study was approved by the Ethics Committee of the China‐Japan Friendship Hospital. All procedures strictly adhered to the ethical principles of the Declaration of Helsinki (1975, revised 1983). Written informed consent was obtained from all participants or their legally authorized representatives for patients with impaired consciousness.

#### 2.1.2 Animals

All experimental protocols were reviewed and approved by the Animal Ethics Committee of the China‐Japan Friendship Hospital. The research complied with the National Health Guidelines for Laboratory Animal Welfare and was conducted in strict accordance with the ARRIVE (Animal Research: Reporting of In Vivo Experiments) guidelines 2.0.

### 2.2 Data Acquisition and Process

HFpEF datasets were retrieved from the NCBI Gene Expression Omnibus (GEO) database (http://www.ncbi.nlm.nih.gov/geo) using the search terms failure with preserved ejection fraction and “HFpEF”. These datasets were then filtered based on specific criteria, including sequencing type (transcriptomics) and sample source (heart tissue). This process led to the identification of GSE108904 (comprising 8 control samples and 8 HFpEF samples) and GSE126062 (comprising 3 control samples and 3 HFpEF samples). The GSE108904 and GSE126062 datasets were generated using the GPL19271 and GPL14746 platforms, respectively.

Primary analysis was conducted on the GSE108904 dataset. Raw data were normalized using the limma package (normalizeBetweenArrays function, “quantile” method). As further analyses were performed within this single dataset, inter‐batch correction was not required.

Independent validation of hub gene expression trends was performed using the GSE126062 dataset. Given the species and platform differences, the datasets were not integrated or batch‐corrected.

A total of 1140 genes related to mitochondria were extracted from the Molecular Signatures Database (MSigDB, http://software.broadinstitute.org/gsea/msigdb).

### 2.3 Identification of Differentially Expressed Genes (DEGs) and Functional Enrichment Analysis

Principal component analysis (PCA) was performed on the normalized expression matrix to assess overall transcriptomic variation, and the number of principal components for downstream assessment was determined based on the elbow point in the scree plot. DEGs were then identified from the GSE108904 dataset using the limmaR package (version 3.56.2). A linear model was fitted to the log2‐transformed expression data, and empirical Bayes moderation was applied to estimate gene‐wise variances. Genes with an adjusted *p*‐value (Benjamini‐Hochberg method) < 0.05 and an absolute log2 fold change (|log2FC | ) > 1.5 were considered statistically significant. The results were visualized with a volcano plot (generated using ggplot2, version 3.4.4) and a clustered heatmap of the top 50 DEGs (by adjusted p‐value) using the pheatmap package (version 1.0.12).

To explore the biological functions and pathways associated with the DEGs, Gene Ontology (GO) and Kyoto Encyclopedia of Genes and Genomes (KEGG) enrichment analyses were performed. Enrichment was conducted using the clusterProfilerR package (version 4.8.3). Terms with an adjusted p‐value (FDR) < 0.05 were considered significantly enriched. Results were visualized using dot plots and chord diagrams. Additionally, Gene Set Enrichment Analysis (GSEA) was performed using the same package against the Hallmark gene set collection (h. all.v2023.2. Hs. symbols. gmt) from the Molecular Signatures Database (MSigDB). Gene sets with a normalized enrichment score (NES) absolute value > 1 and a false discovery rate (FDR) *q*‐value < 0.25 were deemed significant.

### 2.4 Construction of Weighted Gene Co‐Expression Networks

Weighted Gene Co‐expression Network Analysis (WGCNA) was conducted to identify gene modules associated with HFpEF. A soft‐thresholding power (β) of 7 was selected to achieve a scale‐free network topology. Using the resulting adjacency matrix, a topological overlap matrix (TOM) was constructed, followed by hierarchical clustering to detect gene modules. Key parameters for module detection included a mergeCutHeight of 0.25 and a minModuleSize of 30. The module eigengene (ME), defined as the first principal component of each module, was calculated. The correlation between each ME and the HFpEF phenotype was assessed, and the module (the “blue” module) exhibiting the highest absolute correlation coefficient (Pearson's *r* = −0.96, *p* = 1.8e‐9) was designated as the key disease‐associated module for downstream analysis. The WGCNA pipeline was executed using the Sangerbox portal (https://vip.sangerbox.com/).

### 2.5 Identification of Hub Mito‐DEGs

Genes linked to diastolic heart failure were discovered in the Comparative Toxicogenomics Database (CTD) (http://ctdbase.org/) and GeneCards (https://www.genecards.org/). A Venn diagram was employed to demonstrate the intersection among various gene categories, encompassing DEGs, genes closely associated with HFpEF through WGCNA, mitochondrial genes, and diastolic HF‐related genes.

### 2.6 Visualization of Strcture and Protein–Protein Interactions (PPI) of Hub Mito‐DEGs

The molecular structures of the hub Mito‐DEGs were visualized through SWISS‐MODEL (https://swissmodel.expasy.org/). Subsequently, the hub Mito‐DEGs were subjected to PPI analysis using the STRING database (https://string‐db.org/).

### 2.7 Immune Infiltration Analysis

The relative abundance of 36 immune cell types in each sample was evaluated using the ImmuCellAI web portal (http://bioinfo.life.hust.edu.cn/ImmuCellAI/) with default parameters. This tool deconvolutes the immune cell composition based on gene expression signatures. To define distinct cellular subsets within the deconvolution results, a clustering analysis was performed with a resolution parameter set to 0.8. Differences in immune cell infiltration between the Control and HFpEF groups were statistically assessed. Spearman's rank correlation analysis was employed to investigate the relationships between the expression levels of the hub mitochondrial genes (EFHD1, CHCHD1) and the estimated abundance of each immune cell type. The results were visualized with a correlation heatmap using the “corrplot” R package and with violin plots comparing immune cell proportions between groups using the “ggpubr” package.

### 2.8 Prediction of the miRNAs Network of Hub Genes

MiRNAs associated with the hub genes were identified by querying the miRWalk database (http://mirwalk.umm.uni‐heidelberg.de/) and miRDB (https://mirdb.org/). The common miRNAs were determined by intersecting the results from both databases.

### 2.9 Drug Prediction Analysis

CTD was utilized to forecast potential therapeutic agents for the hub Mito‐DEGs.

### 2.10 Validation of Hub Mito‐DEGs in GSE126062 Database

The GSE126062 dataset was employed as a validation database to confirm the expression of the hub Mito‐DEGs using the Wilcoxon test method.

### 2.11 Isolation of Human Peripheral Blood Mononuclear Cells (PBMCs)

PBMCs samples were collected from a cohort of 30 patients diagnosed with HFpEF and 30 healthy controls (HC) devoid of cardiovascular ailments. The diagnosis of HFpEF was based on clinical symptoms, including dyspnea, reduced physical activity, and fluid accumulation, in conjunction with a preserved ejection fraction (EF ≥ 50%) and diastolic dysfunction observed on a transthoracic echocardiogram. PBMCs were extracted from 5 mL of peripheral venous blood using a Ficoll‐Paque solution (TBD, LDS1075) following established protocols [16].

### 2.12 Construction of HFpEF Mouse Models

The experimental procedures were approved by the Institutional Animal Use and Care Committees of China‐Japan Friendship Hospital (No. ZRDWLL240029) and were conducted following NIH guidelines. Adult male C57BL/6 J mice were purchased from Cyagen Biotechnology Co. Ltd. and acclimated for 10 days with ad libitum access to diet and water under controlled conditions. The mice were randomly assigned to either the Control or HFpEF group. Control mice received a standard diet (providing 10% kcal from fat, 20% from protein, and 70% from carbohydrate; D12450B, Research Diets Inc.) and regular drinking water. Mice in the HFpEF group were fed a high‐fat diet (providing 60% kcal from fat, 20% from protein, and 20% from carbohydrate; D12492, Research Diets Inc.) and drinking water supplemented with 0.5 g/L N(ω)‐nitro‐l‐arginine methyl ester (l‐NAME; Sigma‐Aldrich) for 15 weeks. This “two‐hit” approach was selected over acute injury models (e.g., isoproterenol) because it effectively induces the key comorbidities, including insulin resistance, vascular dysfunction, and low‐grade inflammation, that drive the progressive pathophysiology of human HFpEF.

### 2.13 Cell Lines and Culture

The immortalized bone marrow‐derived macrophages (iBMDMs) cell line was obtained from CellVerse Biotechnology Co. Ltd, China. The cells were cultured in DMEM medium supplemented with 10% FBS and 1% antibiotics (100 IU penicillin, 100 μg/mL streptomycin) at 37°C in a humidified 5% CO_2_ incubator. The cells were seeded in six‐well plates at a density of 3 × 106 cells/well. In the PBS or LPS groups, the cells were exposed to an equivalent volume of PBS or lipopolysaccharide (LPS, 1 μg/mL) for 12 h, respectively.

### 2.14 Total RNA Isolation and RT‐qPCR

Total RNA was isolated from human PBMCs, heart tissues, and iBMDMs using the RNAex Pro RNA Kit (Accurate Biotechnology, China). Subsequently, first‐strand cDNA was generated from equivalent quantities of total RNA employing the Revert Aid First Strand cDNA Synthesis Kit (Thermo Fisher Scientific, USA). Quantitative real‐time polymerase chain reaction (qPCR) was performed using SYBR Green (CWBIO, China) on a CFX Connect Real‐Time PCR Detection System (Applied Biosystems, USA). The relative mRNA expression levels of EFHD1 and CHCHD1 were standardized to β‐actin and determined utilizing the ∆∆Ct method. The specific primer sequences utilized are detailed in Table S1.

### 2.15 Protein Extraction and Western Blotting

Total protein was extracted from iBMDMs using RIPA buffer (Beyotime, China) supplemented with 10% proteinase inhibitor (Beyotime, China). The proteins were subsequently separated through 10% SDS‐PAGE. Protein ladder (Thermo Fisher, #26616) was used. Primary antibodies, such as β‐actin (dilution 1:5000, Proteintech) and EFHD1 (dilution 1:1000, Abmart), were left to incubate on PVDF membranes overnight at 4°C. Following this, the membranes underwent incubation with secondary antibodies for 1 h at room temperature and were then detected using the ECL system.

### 2.16 Pathological Staining

Hearts were dissected, fixed in 4% paraformaldehyde overnight, embedded in paraffin, and sectioned into 4 µm‐thick slices. Subsequently, the sections underwent dewaxing, rehydration, and staining with hematoxylin‐eosin (H&E) and Masson's trichrome. Histopathological analysis was performed on the H&E‐stained sections, whereas the Masson's trichrome‐stained sections were utilized for the quantification of collagen content. The slides were examined under an optical microscope after dehydration and mounting.

### 2.17 Immunohistochemistry

The sections underwent deparaffinization, antigen retrieval with citrate buffer (pH 6.0), and blocking with normal goat serum (CST, 5425). Subsequently, they were incubated with primary antibodies targeting EFHD1 (dilution 1:100, Abmart) and CHCHD1 (dilution 1:500, Proteintech), followed by secondary antibody conjugates (dilution 1:5000, Proteintech, China). The slides were counterstained with hematoxylin and examined under a light microscope.

### 2.18 Statistical Analysis

Data are presented as mean ± standard deviation (SD) for normally distributed variables or median (interquartile range) for non‐normally distributed variables. Normality was assessed using the Shapiro‐Wilk test. Comparisons between two groups were performed using the unpaired Student's *t*‐test for normally distributed data or the Mann–Whitney *U* test for non‐normally distributed data, as appropriate. Associations between continuous variables were assessed using Spearman's rank correlation coefficient. Categorical variables were compared using the Chi‐square test or Fisher's exact test, as appropriate. A two‐tailed *P*‐value of < 0.05 was considered statistically significant for all analyses. All statistical analyses were performed using GraphPad Prism version 9.0 (GraphPad Software, San Diego, CA, USA).

## 3 Results

### 3.1 Identification of DEGs and Functional Enrichment Analysis

**Figure 1:**
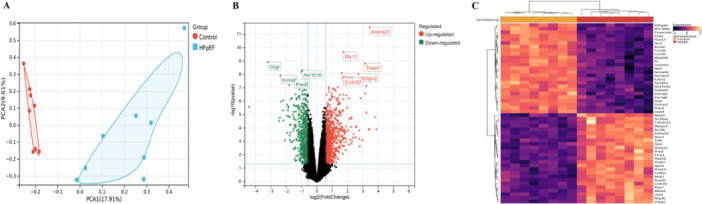
Transcriptomic profiling identifies distinct molecular signatures in HFpEF in GSE108904. (A) Principal component analysis (PCA) demonstrates clear separation between Control (blue, *n* = 8) and HFpEF (red, *n* = 8) groups based on global gene expression patterns. Principal components 1 and 2 explain 17.91% and 8.61% of the total variance, respectively. (B) Volcano plot of differentially expressed genes (DEGs) with significance thresholds set at |log₂(fold change)| > 1.5 and *p*‐value < 0.05. Red points indicate significantly upregulated genes, green points indicate downregulated genes, and gray points represent non‐significant genes. Key annotated genes highlight prominent expression changes. (C) Hierarchical clustering heatmap of DEGs shows distinct expression patterns between Control and HFpEF samples. Rows represent genes, columns represent samples, and color intensity (purple to yellow) reflects normalized expression levels (low to high).

Principal component analysis (PCA) of the GSE108904 dataset demonstrated clear separation between the HFpEF and Control groups, confirming group comparability prior to differential analysis (Figure 1A). Differential expression analysis identified 1279 DEGs, comprising 719 significantly up‐regulated and 560 significantly down‐regulated genes (|log2(FoldChange)| > 1.5, *p*‐value < 0.05), as visualized by volcano plot and heatmap (Figure 1B, C).

**Figure 2:**
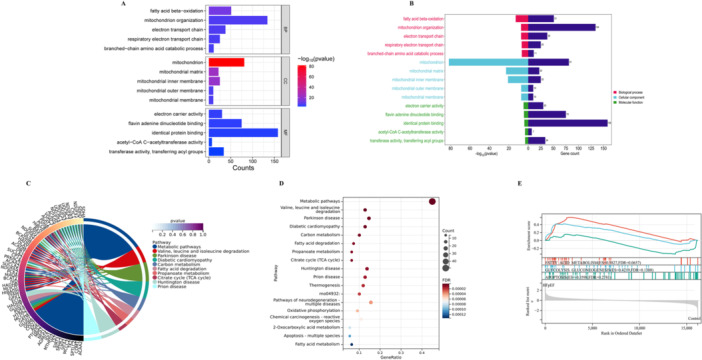
Functional enrichment analysis reveals distinct biological pathways in HFpEF. (A) Gene Ontology (GO) term analysis of differentially expressed genes shows significantly enriched molecular functions and biological processes. Bars are colored by functional category (blue: molecular function, green: biological process) with length representing gene count. The adjacent color scale indicates statistical significance (‐log₁₀(*P*‐value)), with warmer colors (red) denoting higher significance. (B) Comprehensive GO enrichment analysis displays the top enriched terms across three ontologies: biological processes (red), cellular components (cyan), and molecular functions (green). (C) Chord diagram of pathway‐gene interactions illustrates relationships between significant genes (outer circle) and enriched pathways (inner colored segments). Ribbon connections are weighted by association strength and colored according to statistical significance (blue to red gradient representing *P*‐value magnitude). (D) KEGG pathway scatter plot visualizes pathway enrichment results using three dimensions: gene ratio (*x*‐axis), pathway term (*y*‐axis), point size (gene count), and color (false discovery rate, FDR). (E) Gene Set Enrichment Analysis (GSEA) profile compares enrichment patterns between HFpEF (red) and control (gray) groups. The mountain plot shows enrichment scores (*y*‐axis) across gene rank positions (*x*‐axis), with leading‐edge genes marked above each plot.

The enriched GO terms were categorized into BP, CC, and MF, with major terms included fatty acid beta−oxidation, mitochondrion organization, electron transport chain, and branched−chain amino acid catabolic process, among others (Figure 2A, B). The most enriched KEGG pathways associated with the DEGs included pathways such as carbon metabolism, insulin secretion, propanoate metabolism, citrate cycle (TCA cycle), and oxidative phosphorylation, and apoptosis, among others (Figure 2C, D).

GSEA results identified significant enrichment in metabolic pathways, including “FATTY_ACID_METABOLISM” (ES = 0.5827, FDR = 0.1308) and “GLYCOLYSIS_GLUCONEOGENESIS” (positive enrichment: ES = 0.4219, NES = 0.4760; negative enrichment: ES = −0.3598, NES = 0.2781) (Figure 2E).

### 3.2 Construction of Gene Co‐Expression Network and Identification of the Modules

**Figure 3:**
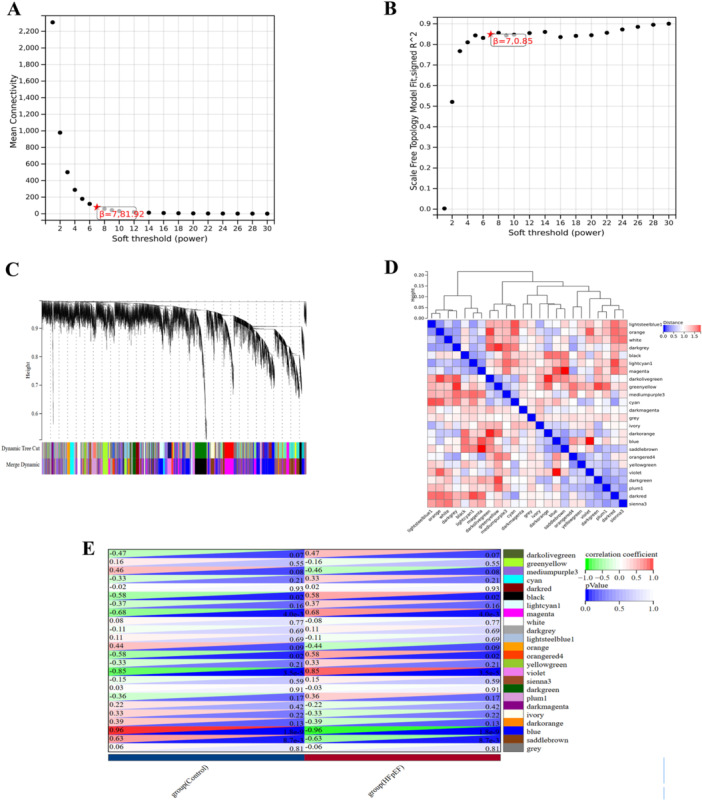
Construction of weighted gene co‐expression network and module identification in HFpEF. (A) Analysis of network topology for different soft‐thresholding powers. The power value β = 7 was selected as the optimal soft threshold to achieve a scale‐free network. (B) Scale‐free topology fit index (R²) plotted against soft‐thresholding powers. The chosen threshold (β = 7) yielded a scale‐free fit index of R² = 0.85, indicating acceptable network connectivity. (C) Hierarchical clustering dendrogram of genes based on topological overlap. Each branch represents a gene, and color bands below denote the module assignments obtained from dynamic tree cutting. (D) Module‐module correlation heatmap. Colors represent the Pearson correlation coefficients between module eigengenes, showing the degree of co‐expression similarity among distinct modules. (E) Module‐trait relationship heatmap. Rows represent module eigengenes, and columns represent HFpEF clinical traits. Color intensity indicates the correlation coefficient (red: positive correlation; blue: negative correlation). The blue module showed the strongest negative correlation with HFpEF phenotypes. HFpEF, heart failure with preserved ejection fraction.

To identify functionally coherent gene groups associated with HFpEF, a weighted gene co‐expression network was constructed using a soft‐thresholding power (β) of 7, achieving a scale‐free topology (R² = 0.85) (Figure 3A, B). Hierarchical clustering identified 24 distinct co‐expression modules (Figure 3C), with their inter‐correlations shown in Figure 3D. Correlation analysis between each module's eigengene and the HFpEF phenotype revealed that the blue module (483 genes) had the strongest negative association (R = −0.96, *P* = 1.8e–9; Figure 3E). This module is enriched for genes involved in mitochondrial bioenergetics, such as electron transport chain components, and immune‐inflammatory regulation. Given that these processes represent two core pathological pillars of HFpEF, the blue module was selected for downstream analysis as a key gene set capturing the critical interplay between metabolic dysfunction and inflammation.

### 3.3 Identification of Hub Mito‐DEGs and Visualization of Molecular Structure and PPI Network Analysis of Hub Mito‐DEGs

**Figure 4:**
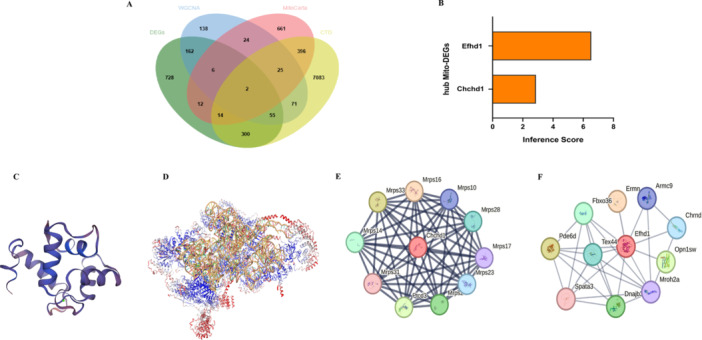
Identification of hub mitochondrial differentially expressed genes (Mito‐DEGs) and structural characterization. (A) Venn diagram illustrating the intersection of genes from four distinct sources: differentially expressed genes (DEGs) between HFpEF and control groups, mitochondrial‐localized genes from MitoCarta 3.0, HFpEF‐associated genes identified by weighted correlation network analysis (WGCNA), and diastolic heart failure‐related genes from the Comparative Toxicogenomics Database (CTD). The overlapping region reveals candidate hub Mito‐DEGs functionally linked to HFpEF pathogenesis. (B) Inference scores of hub Mito‐DEGs (EFHD1 and CHCHD1) regarding their association with diastolic heart failure, as quantified by the CTD database. Higher scores indicate stronger evidence‐based relationships with disease pathophysiology. (C, D) Molecular structural models of EFHD1 (C) and CHCHD1 (D) generated by homology modeling using the SWISS‐MODEL database. Structures highlight functional domains including EF‐hand calcium‐binding motifs in EFHD1 and coiled‐coil helix domains in CHCHD1. (E, F) Protein–protein interaction (PPI) networks of EFHD1 (E) and CHCHD1 (F) constructed using the STRING database. Nodes represent proteins, edges indicate functional or physical interactions, and network layout reflects functional modularity and biological connectivity. EFHD1, EF‐hand domain‐containing protein D1; CHCHD1, coiled‐coil‐helix‐coiled‐coil‐helix domain containing 1; HFpEF, heart failure with preserved ejection fraction.

Mito‐DEGs were identified by intersecting four gene sets: the DEGs, genes from the HFpEF‐associated WGCNA module, 1140 mitochondria‐localized genes from MitoCarta3.0, and 7946 diastolic heart failure‐related genes from the CTD database (Figure 4A). The two resulting hub genes, EFHD1 and CHCHD1, showed high inference scores for association with diastolic heart failure in CTD (Figure 4B). Their molecular structures were modeled using SWISS‐MODEL (Figure 4C, D), and their protein–protein interaction networks were constructed with STRING (Figure 4E, F).

### 3.4 Immune Infiltration in HFpEF and Correlation Between Hub Mito‐DEGs and Immune Cells

**Figure 5:**
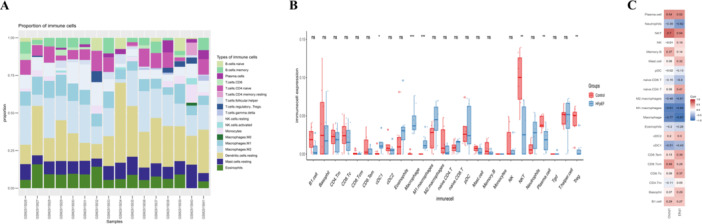
Immune infiltration landscape and its association with mitochondrial genes in HFpEF. (A) Stacked bar chart displaying the relative proportions of 36 immune cell types across Control and HFpEF samples. Each column represents an individual sample, with colored segments indicating distinct immune cell populations quantified by the ImmuCellAI algorithm. (B) Violin plots comparing immune cell distribution between the Control and HFpEF groups. (C) Correlation heatmap illustrating Spearman correlations between hub Mito‐DEGs (EFHD1 and CHCHD1) and immune cell abundances. Red indicates positive correlations, blue indicates negative correlations. EFHD1, EF‐hand domain‐containing protein D1; CHCHD1, coiled‐coil‐helix‐coiled‐coil‐helix domain containing 1; HFpEF, heart failure with preserved ejection fraction. *p < 0.05, **p < 0.01, ***p < 0.0010.

Furthermore, Spearman's rank correlation coefficient was employed to investigate the potential relationship between hub Mito‐DEGs and immune cells (Figure 5A, B). EFHD1 and CHCHD1 exhibited positive correlations with Plasma cells, NKT cells, Memory B cells, CD8 Tcm cells, and B1 cells, while showing negative associations with Neutrophils, Macrophages, M1 Macrophages, M2 Macrophages, and Eosinophils (Figure 5C).

### 3.5 Prediction of miRNAs Targeting Hub Genes

**Figure 6:**
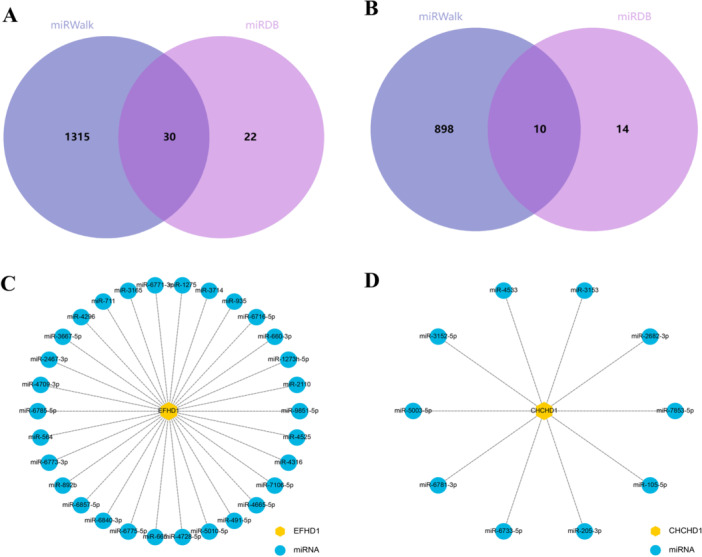
MiRNA regulatory network establishment based on hub Mito‐DEGs. (A) Venn diagram showing intersection miRNA target EFHD1 between miRWalk and miRDB databases. (B) Venn diagram showing intersection miRNA target CHCHD1 between miRWalk and miRDB databases. (C) Visualization of the common miRNAs target EFHD1. (D) Visualization of the common miRNAs target CHCHD1. miRNA, microRNAs. DEGs, differentially expressed genes; EFHD1, EF‐hand domain‐containing protein D1; CHCHD1, coiled‐coil‐helix‐coiled‐coil‐helix domain containing 1.

The miRNAs targeting the hub Mito‐genes were screened in the miRWalk and miRDB databases. Figure 6A, B showed that 1348 miRNAs in the miRWalk database and 52 in the miRDB database targeting EFHD1, while 908 miRNAs in miRWalk and 24 in miRDB targeting CHCHD1. Subsequently, the common miRNAs were visualized after creating the intersection Venn diagram (Figure 6C, D).

### 3.6 Drug Prediction Analysis of the Key Genes

Regarding the drug prediction analysis of the hub Mito‐genes, the CTD database was utilized to predict potential therapeutic agents for the key genes, with the results presented in Table S2. A total of 13 drugs were predicted for EFHD1 and 2 for CHCHD1.

### 3.7 Validation of Hub Mito‐DEGs in GSE126062 Database

**Figure 7:**
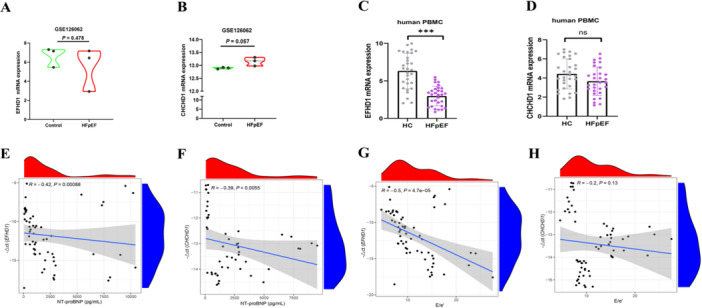
Multi‐level validation of hub mitochondrial gene expression and clinical correlations in HFpEF. (A, B) Expression analysis of EFHD1 (A) and CHCHD1 (B) mRNA in the independent validation dataset GSE126062 (Control, *n* = 3; HFpEF, *n* = 3), statistical significance was determined by Student's *t*‐test, with data represented as mean ± SD. (C, D) Validation of EFHD1 (C) and CHCHD1 (D) mRNA expression in peripheral blood mononuclear cells (PBMCs) isolated from healthy controls (HC, *n* = 30) and HFpEF patients (*n* = 30), data are presented as median (IQR). Statistical significance was assessed by the Mann–Whitney *U* test (*p < 0.05, **p < 0.01, ***p < 0.001 vs. HC group). (E, F) Spearman correlation analysis between EFHD1 (E) and CHCHD1 (F) gene expression (ΔΔCt values) and NT‐proBNP levels. (G, H) Correlation analysis between EFHD1 (G) and CHCHD1 (H) gene expression (ΔΔCt values) and E/e’. EFHD1, EF‐hand domain‐containing protein D1; CHCHD1, coiled‐coil‐helix‐coiled‐coil‐helix domain containing 1; NT‐proBNP, N‐terminal pro‐B‐type natriuretic peptide; HFpEF, heart failure with preserved ejection fraction.

To independently validate the bioinformatics findings, we analyzed the expression of EFHD1 and CHCHD1 in a separate murine HFpEF dataset, GSE126062. In this dataset, EFHD1exhibited a downward trend and CHCHD1 an upward trend in the HFpEF group compared to controls; these changes did not reach statistical significance (Figure 7A, B), likely due to the limited sample size (*n* = 3 per group).

### 3.8 Clinical and Experimental Validations of Hub Mito‐Genes In Vivo and In Vitro

Compared to healthy controls (HC, *n* = 30), HFpEF patients (*n* = 30) exhibited higher BMI, impaired glucose metabolism, and elevated diastolic dysfunction markers (Table 1). Their PBMCs showed a pronounced pro‐inflammatory state, with significant upregulation of IL‐1β, TNF‐α, and NLRP3 (Figure S1A–C). Within this inflammatory milieu, EFHD1 expression was markedly reduced, while CHCHD1 levels showed no significant change (Figure 7C, D). EFHD1 expression correlated negatively with both NT‐proBNP levels (R = −0.42, *p* = 0.00088) and the E/e’ ratio (R = −0.5, *p* = 4.7e–05). CHCHD1 correlated negatively only with NT‐proBNP (R = −0.39, *p* = 0.0055) (Figure 7E–H).

**Table 1:** Characteristics of the study population.

Characteristics	HC (*n* = 30)	HFpEF (*n* = 30)	*p* value
Demographic feature			
Age (years)	60.83 ± 6.845	62.20 ± 12.78	0.467
Sex (male, %)	17 (58.3)	20 (65.0)	0.523
BMI (kg/m^2^)	21.80 ± 2.72	24.44 ± 4.34	< 0.0001
Vital signs			
Systolic blood pressure, mmHg	117.8 ± 22.5	122.5 ± 21.8	0.177
Diastolic blood pressure, mmHg	73.8 ± 12.1	86.2 ± 13.7	0.085
Clinical features			
TC (mmol/L)	4.89 (3.06, 7.26)	5.38 (2.09, 90.00)	0.379
TG (mmol/L)	2.09 (0.38, 21)	3.71 (0.36, 31.24)	0.037
LDL‐C (mmol/L)	2.81 ± 0.79	2.90 ± 0.82	0.004
HDL‐C (mmol/L)	1.30 ± 0.49	1.04 ± 0.27	0.418
FBG (mmol/L)	5.44 (4.31, 11.55)	9.35 (2.94, 29.59)	< 0.0001
HbA1C (%)	5.63 ± 0.62	6.93 ± 1.91	< 0.0001
ALT (U/L)	23.37 (6, 122)	38.22 (4, 402)	0.100
AST (U/L)	20.42 (11, 43)	36.05 (7, 306)	0.067
NT‐proBNP (ng/L)	83.63 (9, 319)	5690 (26, 35000)	< 0.0001
hs‐CRP (mg/L)	5.64 (1.05, 9.98)	24.15 (0.79, 107.52)	< 0.0001
Echocardiogram indexes			
LVEF (%)	61.62 ± 6.57	59.95 ± 5.97	0.149
FS (%)	34.48 ± 4.22	34.02 ± 4.47	0.575
E/A	1.21 ± 0.38	0.86 ± 0.37	< 0.0001
IVST (mm)	8.97 ± 1.33	10.91 ± 2.29	< 0.0001
LVPW (mm)	7.92 ± 1.46	9.32 ± 1.95	< 0.0001
E/e'	9.73 (6.67, 14.03)	12.43 (5.46, 27.50)	< 0.0001

Abbreviations: ALT, alanine aminotransferase; AST, aspartate aminotransferase; BMI, body mass index; Cr, creatinine; eGFR, estimating glomerular filtration rate; FBG, fasting bllod glucose; FS, Left ventricular fractional shortening; HBA1c, glycosylated hemoglobin; HC, healthy controls; HDL‐C, high‐density lipoprotein cholesterol; hs‐CRP, high‐sensitivity C‐reactive protein; LDL‐C, low‐density lipoprotein cholesterol; LVEF, left ventricular ejection fraction; left ventricular end‐diastolic internal diameter; NT‐proBNP, N‐terminal pro‐B‐type natriuretic peptide; TC, total cholesterol; TG, triglycerides.

**Figure 8:**
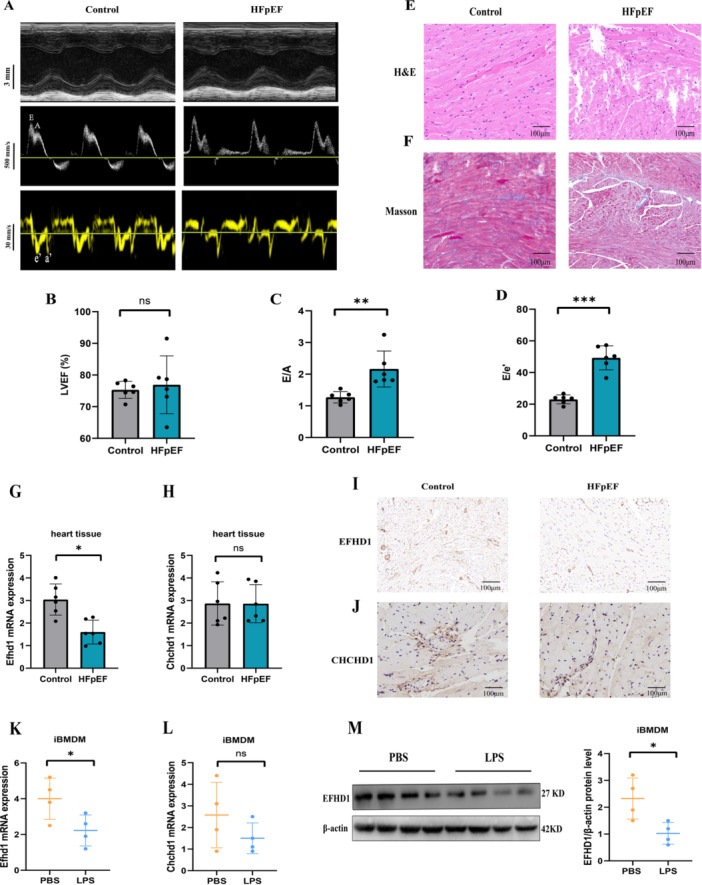
Multi‐level analysis of mitochondrial gene expression in cardiac tissues and macrophages in a HFpEF model. (A)Representative echocardiographic images and tissue Doppler waveforms from Control and HFpEF mice. Scale bars: 3 mm (top), 500 mm/s (middle), 30 mm/s (bottom). (B–D) Quantitative analysis of echocardiographic parameters (*n* = 6): (B) Left ventricular ejection fraction (LVEF), (C) E/A ratio, and (D) E/e’ ratio. (E, F) Histopathological examination of cardiac tissues by hematoxylin and eosin (H&E) and Masson's trichrome staining. H&E staining displays cardiomyocyte morphology and inflammatory cell infiltration, while Masson's staining highlights collagen deposition (blue areas), indicating increased fibrosis in HFpEF hearts compared to Controls. Scale bars: 100 μm. (G, H) mRNA expression analysis of Efhd1 (G) and Chchd1 (H) in cardiac tissues from Control and HFpEF groups (*n* = 6 per group). (I, J) Immunohistochemical (IHC) staining for EFHD1 (I) and CHCHD1(J) proteins in cardiac tissues. Scale bars: 100 μm. (K, L) mRNA expression analysis of Efhd1 (K) and Chchd1 (L) in immortalized bone marrow‐derived macrophages (iBMDMs) stimulated with phosphate‐buffered saline (PBS, control) or lipopolysaccharide (LPS) to induce inflammation (*n* = 4 per group). (M) Western blot analysis of EFHD1 protein expression in iBMDMs treated with PBS or LPS (*n* = 4 per group). Statistical significance was determined by Student's *t*‐test, with data represented as mean ± SD (*p < 0.05, **p < 0.01, ***p < 0.001 vs. Control group). EFHD1, EF‐hand domain‐containing protein D1; CHCHD1, coiled‐coil‐helix‐coiled‐coil‐helix domain containing 1; HFpEF, heart failure with preserved ejection fraction.

HFpEF mice showed preserved LVEF but significantly impaired diastolic function, evidenced by increased E/A and E/e’ ratios, alongside enhanced myocardial fibrosis (Figure 8A–F). Cardiac tissue exhibited elevated expression of pro‐inflammatory mediators (Il‐1β, Tnf‐α, Nlrp3) (Figure S1D–F). Consistent with human data, Efhd1 mRNA was significantly downregulated in HFpEF hearts, whereas Chchd1 expression was unchanged (Figure 8G, H). Immunohistochemistry confirmed reduced EFHD1 and unchanged CHCHD1 protein levels (Figure 8I, J).

In LPS‐stimulated immortalized bone marrow‐derived macrophages (iBMDMs), EFHD1 expression was significantly decreased at both mRNA and protein levels. CHCHD1 showed a non‐significant decreasing trend (Figure 8K–M).

## 4 Discussion

HFpEF has become the predominant form of heart failure worldwide. However, its pathophysiology is not fully understood, and effective targeted therapies are still lacking [17]. Our study identifies EFHD1 as a key mitochondrial gene potentially linking metabolic dysfunction and immune dysregulation in HFpEF, while CHCHD1 also showed intriguing associations warranting further investigation. These findings offer new mechanistic insight into how mitochondrial impairment may contribute to disease progression and highlight immunometabolic crosstalk as a promising therapeutic avenue.

Consistent with previous studies [18, 19], we observed concordant up‐regulation of NLRP3, IL‐1β, and TNF‐α in both patient PBMCs and HFpEF mouse myocardium. This alignment supports the HFD/l‐NAME model as a reflection of the low‐grade, inflammasome‐related inflammation seen in human HFpEF and provides a basis for examining mitochondrial–immune interplay in this setting.

Mitochondrial dysfunction is increasingly recognized as a central feature of HFpEF, particularly given the heart's high energy demand and reliance on oxidative phosphorylation (OXPHOS) [20]. Our bioinformatics analyses revealed profound alterations in mitochondrial pathways, with distinct transcriptomic profiles distinguishing control and HFpEF groups. Enrichment analyses further demonstrated significant involvement of key biological processes, including fatty acid β‐oxidation, citrate cycle, and OXPHOS. We further identified downregulation of EFHD1 and CHCHD1 as key mitochondrial alterations in HFpEF, with both genes playing critical roles in maintaining mitochondrial integrity and energy metabolism.

EFHD1 contains two Ca²⁺‐binding EF‐hand motifs and a coiled‐coil region that mediates protein–protein interactions [21]. The protein is chiefly mitochondrial, with evidence pointing to localization at the inner membrane [22]. Clinically, variations in EFHD1 have been associated with circulating liver enzyme levels in humans, suggesting a role in systemic metabolic regulation [22]. Under conditions of mitochondrial oxidative stress, such as downregulation of superoxide dismutase 2 (SOD2), EFHD1 expression is frequently upregulated, indicating a potential compensatory response [22]. Further supporting its metabolic role, EFHD1 inhibition in pro‐B immune cells leads to altered mitochondrial function and a shift toward glycolytic metabolism [22]. Notably, studies by Eberhardt et al. demonstrated that EFHD1 ablation suppresses cardiac mitoflash activity and exerts a protective effect on cardiomyocytes under ischemic conditions [22], highlighting its context‐dependent function in stress adaptation.

CHCHD1 is a mitochondrial ribosomal protein essential for the biosynthesis of key subunits within OXPHOS complexes, with mutations frequently leading to OXPHOS impairments that underscore its critical role in cellular energy production [23, 24]. However, its role likely extends beyond bioenergetics. CHCHD1's extensive interactome, involving over 50 proteins regulating apoptosis, protein import, and membrane dynamics, positions it as a critical hub for mitochondrial quality control [25]. Its dysregulation could impair the organelle's ability to respond to stress, leading to the release of mitochondrial DAMPs (damage‐associated molecular patterns) that further fuel the NLRP3 inflammasome and the chronic inflammatory state we observed [26, 27]. This integrative perspective suggests that CHCHD1 loss contributes to HFpEF not only by reducing ATP output but also by fostering a pro‐apoptotic and pro‐inflammatory microenvironment, thereby promoting cardiomyocyte loss and fibrotic remodeling.

In the validation dataset GSE126062, both EFHD1 and CHCHD1 showed consistent downregulation trends, though statistical significance was not achieved. Analysis of patient‐derived PBMCs revealed significantly reduced EFHD1 expression in HFpEF subjects compared to healthy controls, while CHCHD1 exhibited a non‐significant decreasing tendency. Notably, EFHD1 expression demonstrated a strong inverse correlation with both NT‐proBNP levels and the E/e’ ratio, whereas CHCHD1 correlated significantly only with NT‐proBNP. Consistent with human findings, EFHD1 was significantly downregulated in cardiac tissues of HFpEF mice and in LPS‐stimulated iBMDMs, confirming its relevance across species and model systems.

Cardiac immunopathological analysis in HFpEF revealed significant disruption of immunoregulatory balance. Anti‐inflammatory Tregs were markedly reduced, impairing inflammatory control and potentially accelerating fibrosis [28, 29], while altered NK cell distribution implicated their role in ventricular remodeling [30]. Pro‐inflammatory subsets showed substantial expansion. cDC1 dendritic cells promoted T‐cell activation through antigen presentation [31]. Neutrophils mediated cardiomyocyte damage via myeloperoxidase release [32]. Dominant M1 macrophage polarization reinforced a pro‐inflammatory milieu [33]. Although eosinophils typically constitute 1–3% of circulating leukocytes, their cardiac accumulation promoted thrombosis and fibrosis [34].

This systemic pro‐inflammatory shift exacerbated myocardial fibrosis, impaired diastolic function, and established a vicious cycle of tissue damage. The inverse correlation between EFHD1/CHCHD1 and macrophage infiltration suggests mitochondrial‐immune crosstalk in HFpEF. Immune‐targeted strategies, including Treg enhancement and macrophage modulation, may offer therapeutic promise [35].

The prediction of miRNAs targeting these key mitochondrial genes adds another layer of regulatory complexity to our findings. The identification of numerous candidate miRNAs for both EFHD1 and CHCHD1 suggests sophisticated post‐transcriptional regulation of these mitochondrial proteins, potentially contributing to the fine‐tuning of their expression in response to metabolic stress conditions. This regulatory network may represent an adaptive mechanism that becomes compromised in HFpEF pathogenesis.

Similarly, the drug prediction analysis revealing multiple potential therapeutic agents for EFHD1 and CHCHD1 highlights the translational potential of our findings. These computational predictions provide valuable starting points for developing targeted interventions aimed at restoring mitochondrial function in HFpEF.

Several limitations of our current work should be acknowledged. First, the findings require validation through large‐scale clinical studies to establish their generalizability. Second, the functional roles of the identified hub genes need to be further elucidated through genetic manipulation approaches such as knockout or overexpression models in cellular and animal systems. Third, the precise molecular mechanisms through which these mitochondrial regulators influence HFpEF progression, particularly their roles in mediating cross‐talk between metabolic pathways and immune responses, warrant further investigation. Additionally, while our study focused on macrophage‐specific mechanisms using an established iBMDM model, future investigations employing primary human cardiac cells would provide important insights into cardiomyocyte‐immune interactions in HFpEF.

## 5 Conclusion

In conclusion, our findings position EFHD1 and CHCHD1 as key mediators connecting mitochondrial dysfunction with immune activation in HFpEF, providing new insights into disease mechanisms and potential therapeutic targets for this challenging syndrome.

## Author Contributions

The work presented here was carried out in collaboration among all authors. Yingying Xie, Yanxiang Gao, and Jingang Zheng defined the theme of the study and discussed the analysis, interpretation, and presentation. Haoming He analyzed the data, developed the algorithm, and explained the results. Yingying Xie conducted subsequent experimental verification. Yingying Xie and Haoming He drafted the manuscript. Yike Li and Qiang Chen participated in the collection of relevant data and helped draft the manuscript. All the authors read and approved the final manuscript.

## Ethics Statement

This study was approved by the Ethics Committee of the China–Japan Friendship Hospital. All procedures strictly adhered to the ethical principles of the Declaration of Helsinki (1975, revised 1983).

All experimental protocols were reviewed and approved by the Animal Ethics Committee of the China–Japan Friendship Hospital. The research complied with the National Health Guidelines for Laboratory Animal Welfare and was conducted in strict accordance with the ARRIVE (Animal Research: Reporting of In Vivo Experiments) guidelines 2.0.

## Consent

Written informed consent was obtained from all participants or their legally authorized representatives for patients with impaired consciousness.

## Conflicts of Interest

The authors declare no conflicts of interest.

## Supporting information

Supporting Figure S1

Supporting Table S1

Supporting Table S2

## Data Availability

The GEO datasets used for analysis in this study (GSE108904, and GSE126062) were derived from the Gene Expression Omnibus (https://www.ncbi.nlm.nih.gov/geo/), a publicly available repository. The data generated and/or analysed from human PBMCs are available from the corresponding author on reasonable request.
