# Supplementary material for: Linking Mitochondrial Dysfunction to the Immune Microenvironment in HFpEF: An Integrated Bioinformatics and Experimental Approach
Source: Immun Inflamm Dis. 2026 Apr 22;14(4):e70440. doi: 10.1002/iid3.70440 (PMC13103268; doi:10.1002/iid3.70440)
Supplement: Supplementary file 3 — Supporting Table S2 [file IID3-14-e70440-s003.docx]

| **Gene** | **Primer sequence (forward)** | **Primer sequence (reverse)** |
| --- | --- | --- |
| EFHD1 (human) | GGAGGGTGTCAAAGGTGCCAAG | CGCTTCCGCTCATCTTGCTCAG |
| Efhd1  (mouse) | GAAGGTGTCAGAGGTGCCAAGAAC | GCCTCCTCCTCCCGTTTCCG |
| CHCHD1  (human) | AGCCTGTGCTGAAGCCCAATAAAC | CAAGCCATCATCACCGACATCTCC |
| Chchd1  (mouse) | GGCAACCTGTATCACGGAGATGTC | CCTGAGCCCTGGAAGAACAATCG |
| IL-1β  (human) | CCACCTCCAGGGACAGGATA | TCAACACGCAGGACAGGTAC |
| Il-1β  (mouse) | TCGCAGCAGCACATCAACAAGAG | AGGTCCACGGGAAAGACACAGG |
| TNF-α  (human) | AGCCCTGGTATGAGCCCATCTATC | TCCCAAAGTAGACCTGCCCAGAC |
| Tnf-α  (mouse) | ATGGCCTCCCTCTCATCAGT | AAGGTACAACCCATCGGCTG |
| NLRP3  (human) | GGCAACACTCTCGGAGACAA | GGAAAGATCCCAGCAGCAGT |
| Nlrp3  (mouse) | GCCGTCTACGTCTTCTTCCTTTCC | CATCCGCAGCCAGTGAACAGAG |

**Table S2. Primers’ sequences used in this study.**
